# Quantitative assessment of AD markers using naked eyes: point-of-care testing with paper-based lateral flow immunoassay

**DOI:** 10.1186/s12951-021-01111-z

**Published:** 2021-11-17

**Authors:** Liding Zhang, Xuewei Du, Ying Su, Shiqi Niu, Yanqing Li, Xiaohan Liang, Haiming Luo

**Affiliations:** 1 Britton Chance Center for Biomedical Photonics, Wuhan National Laboratory for Optoelectronics, Huazhong University of Science and Technology, Wuhan, China; 2 MoE Key Laboratory for Biomedical Photonics, School of Engineering Sciences, Huazhong University of Science and Technology, Wuhan, China; 3 Engineering Research Center of Nano-Geomaterials of Ministry of Education, Faculty of Materials Science and Chemistry, China University of Geosciences, Wuhan, China; 4 Department of Neurology, Union Hospital, Tongji Medical College, Huazhong University of Science and Technology, Wuhan, 430022 China; 5 Wuhan, China

**Keywords:** Alzheimer’s disease, Blood, Aβ_42_ monomer, Aβ_42_ oligomer, Magnetic nanoparticles, Gold nanoparticle, Paper-based lateral flow immunoassay

## Abstract

Aβ_42_ is one of the most extensively studied blood and Cerebrospinal fluid (CSF) biomarkers for the diagnosis of symptomatic and prodromal Alzheimer’s disease (AD). Because of the heterogeneity and transient nature of Aβ_42_ oligomers (Aβ_42_Os), the development of technologies for dynamically detecting changes in the blood or CSF levels of Aβ_42_ monomers (Aβ_42_Ms) and Aβ_42_Os is essential for the accurate diagnosis of AD. The currently commonly used Aβ_42_ ELISA test kits usually mis-detected the elevated Aβ_42_Os, leading to incomplete analysis and underestimation of soluble Aβ_42_, resulting in a comprised performance in AD diagnosis. Herein, we developed a dual-target lateral flow immunoassay (dLFI) using anti-Aβ_42_ monoclonal antibodies 1F12 and 2C6 for the rapid and point-of-care detection of Aβ_42_Ms and Aβ_42_Os in blood samples within 30 min for AD diagnosis. By naked eye observation, the visual detection limit of Aβ_42_Ms or/and Aβ_42_Os in dLFI was 154 pg/mL. The test results for dLFI were similar to those observed in the enzyme-linked immunosorbent assay (ELISA). Therefore, this paper-based dLFI provides a practical and rapid method for the on-site detection of two biomarkers in blood or CSF samples without the need for additional expertise or equipment.

**Supplementary Information:**

The online version contains supplementary material available at 10.1186/s12951-021-01111-z.

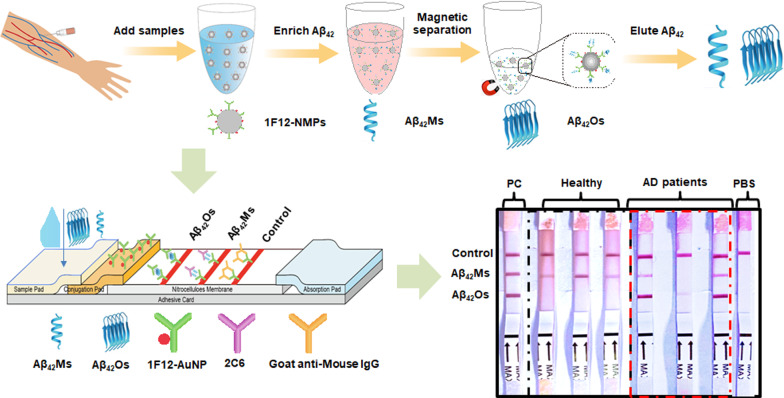

## Introduction

Alzheimer's disease (AD) is a chronic neurodegenerative disorder, which may begin to develop 20–30 years before clinical onset, accompanied by progressive neuropathology, brain atrophy, and ultimately lead to cognitive decline [1–3]. By 2050, the number of AD worldwide will increase from the estimated 50 million reported in 2018 to 152 million [4]. Current treatment strategies for AD are limited to symptom relief [5, 6]. There is an urgent need to develop practical diagnostic tools and conduct large-scale early prevention and screening of high-risk groups to solve the public health crisis caused by AD. Presently, the diagnosis of AD is still mainly based on clinical cognitive assessment and physical examination. Histopathological examination of brain autopsy specimens is the only way to diagnose definite AD. Although the exact mechanism of AD pathogenesis remains elusive, amyloid β (Aβ) has been proposed to be an essential biomarker and therapeutic target for AD [7–9]. To date, magnetic resonance imaging (MRI) for measuring brain volume and neuronal connections, and Aβ- or tau-based positron-emission tomography (PET) for detecting pathological protein deposition in the brain have been used in AD diagnosis [10–13]. Although MRI and PET imaging tools with good diagnostic performances of brain diseases are approved for clinical use, the economic burden of imaging hinders their wide application in the identification of AD [14]. In addition, limited MRI or PET equipment is difficult to meet the growing number of AD patients. Expect for imaging diagnosis tools, it has also developed the detection of cerebrospinal fluid (CSF) biomarkers, such Aβ_1-40_ (Aβ_40_), Aβ_1-42_ (Aβ_42_), and the phosphorylation of tau (p-Tau) for AD diagnosis. Among them, the Aβ-based ELISA methods have been used as a reference for clinical diagnosis [15–17].

Aβ is a 38–43 amino acid polypeptide derived from amyloid precursor protein that is continuously cleaved by β- and γ-secretase. The predominant Aβ subtypes are Aβ_40_ and Aβ_42_, of which Aβ_42_ is easier to form fibrils and has a stronger tendency to aggregate. Increasing evidence has implicated that soluble Aβ_42_ oligomers (Aβ_42_Os) are the most critical toxic species causing AD-related synapse/neuron loss [18, 19] and memory decline [20]. Aβ_42_ monomers (Aβ_42_Ms) can modulate the Aβ self-assembly process to maintain intracellular signal transduction to achieve synaptic plasticity and homeostasis [21]. Therefore, both Aβ_42_Os and Aβ_42_Ms are related to the pathogenesis of AD. Abnormal Aβ_42_ levels can be detected in CSF and amyloid-β PET neuroimaging (amyloid-PET) [22–24]. However, there are some limitations, such as high testing costs, limited equipment, and invasiveness [25]. Compared with CSF measurements that may cause lumbar puncture and back pain, blood-related diagnosis tools are more acceptable due to their ease of collection, less invasiveness, and low cost. Multiple evidence shows that Aβ appearing in human blood [26–28] may be transmitted by peripheral monocytes [29], axonal [30, 31], exosomes [32, 33] from the brain over the blood–brain barrier. Furthermore, changes in the increased β-sheet structure of Aβ in human blood are associated with AD biomarkers in cerebrospinal fluid (CSF) and amyloid-PET [27]. Dynamic monitoring of Aβ_42_Ms and Aβ_42_Os levels in the blood helps to accurately diagnose AD before clinical symptoms appear.

Many attempts have been made to explore new diagnostic tools for several core biomarkers, including enzyme-linked immunosorbent assay (ELISA) [34–36], nanoparticle-based immunoassays [37], electrochemistry [38], surface-enhanced Raman spectroscopy [39, 40], fluorescence [41], and electrochemical biosensors [42, 43]. However, due to the heterogeneity and transient nature of Aβ oligomers, most of these assays not only cannot distinguish between Aβ_42_Os and Aβ_42_Ms but also require equipment or/and expertise. The development of technologies that can dynamically detect changes in the levels of Aβ_42_Ms and Aβ_42_Os in the blood is essential for the accurate diagnosis of AD.

Paper-based lateral flow immunoassay (LFI) has been widely used for the rapid diagnosis of blood biomarkers due to its simplicity, portability, cost-effectiveness, and rapid detection of target biomarkers [44–47]. However, the accuracy and sensitivity of LFIS to a single biomarker still need to be improved, especially in the face of complicated pathological diseases such as AD with multiple biomarkers, including Aβ_42_Ms, Aβ_42_Os, p-Tau^181^, and p-Tau^217^. Currently, diagnostic tools for AD mainly target one biomarker, such as Aβ, p-Tau, or total tau, with compromised sensitivity and specificity. Previous studies reported that the average specificities of AD diagnostic methods based on Aβ, t-tau, or p-tau are 84.1%, 82.3%, and 83%, while their average sensitivities were 79.4%, 80.5%, and 78.3% [48–50]. However, the average specificity and sensitivity of multi-biomarker-based detection methods are over 86% and 83.5%, which are relatively higher than those based on a single biomarker Aβ, p-Tau, or total tau [48–50]. Recently, we screened the preferred antibody pairs 1F12/1F12 and 1F12/2C6 to achieve accurate detection of Aβ_42_Ms and Aβ_42_Os. The strategy used in our previous study for the detection of oligomeric and monomeric Aβ was to use the antibody 1F12 with a single epitope to capture Aβ_42_Ms and Aβ_42_Os and then incubated with the detection antibody 1F12 with the same epitope for detecting only oligomers because oligomers were aggregated by multiple monomers, exposing several identical epitopes that can be recognized by the same detection antibody. While for Aβ_42_Ms, they were only detected by the antibody 2C6 with different epitopes from the capture antibody 1F12 to form a sandwich structure [51]. In addition, our analysis of the levels of Aβ_42_Ms and Aβ_42_Os in brain and blood samples was closely related to the progression of AD, indicating that simultaneous detection of Aβ_42_Ms and Aβ_42_Os may improve the specificity and sensitivity of AD detection [51]. Given that, we look forward to developing a test strip that can accurately detect the levels of Aβ_42_Ms and Aβ_42_Os in the blood to achieve a rapid and accurate diagnosis of AD. In this study, we developed a dual-target lateral flow immunoassay (dLFI) by adopting Aβ_42_-specific monoclonal antibodies 1F12 and 2C6 with unique/overlapping epitopes to create steric hindrance between antibody capture and detection. Herein, 1F12 was labeled with gold nanoparticles (1F12-AuNP) as the capture antibody. Then, 1F12 and 2C6 were used as detection antibodies to be immobilized on nitrocellulose (NC) membrane as two test lines, and goat anti-mouse IgG was immobilized on one end of the NC membranes as a control line. The clinical manifestations of dLFI were tested in blood samples of 5xFAD mice and AD patients. We aimed to develop a multi-objective LFI for rapid and high-performance diagnosis of AD.

## Materials and methods

### Materials

Aβ_42_ (DAEFRHDSGYEVHHQKLVFFAEDVGSNKGAIIGLMVGGVVIA), Aβ_40_ (DAEFRHDSGYEVHHQKLVFFAEDVGSNKGAIIGLMVGGVV), P-Tau^396,404^ (RENAKAKTDHGAEIVYK-[Ser(P)]PVVSGDT[Ser(P)]PRHL), Cis-Tau (KVAVVRpT(5,5-dimethyl-l-proline)PKSPS), and P-Tau^231^ (KVAVVRpTAPKSPS) were custom-synthesized as lyophilized powders by Royo Biotech Co., Ltd (Shanghai, China) with a purity of > 95%. The detailed information of synthesized peptides, including HPLC and mass spectrometry results, are shown in Additional file 1: Fig. S1–3. The Aβ_42_-specific monoclonal antibodies (mAbs) 1F12 and 2C6 were produced in our laboratory, of which the epitope of 1F12 is Aβ_3–9_, while the epitopes of 2C6 are Aβ_3–9_, Aβ_13–19_, Aβ_18–25_, Aβ_29–36_, and Aβ_36–42_ [51]. Trisodium citrate dehydrates (HAuCl_4_) and bovine serum albumin (BSA) were purchased from Sigma Aldrich (St. Louis, MO, USA). NaCl, polyvinyl pyrrolidone (PVP), K-40, sucrose, Casein, NaN_3_, PEG, Tris, MES, and Tween-20 were obtained from Beijing Biotopped Science and Technology Co., Ltd. (Beijing, China). Filter paper and semi-rigid polyvinyl chloride (PVC) sheets were purchased from Jieyi Biological Technology Co., Ltd. (Shanghai, China). Glass fibre membranes and nitrocellulose membranes were obtained from Millipore (Billerica, MA, USA). Goat anti-mouse IgG and protein A resins were purchased from GenScript (Nanjing, China). N-Hydroxysuccinimide (NHS) modified magnetic nanoparticles were obtained from LinkedIn Biotechnology Co., Ltd. (Shanghai, China).

### Preparation of gold nanoparticles (AuNP)

AuNP was synthesized following the HAuCl_4_ reduction scheme with citric acid [52]. Briefly, 99 mL of ultrapure water was added to a purified bottle, and 1 mL of 1% HAuCl_4_·3H_2_O was then added with continuous stirring and heating. The solution was heated until the solution started to boil slightly, and 1 mL of filtered 1% trisodium citrate solution was then added. After heating for a few minutes, the solution gradually changed from colorless to grey, black, purple, and red. The heating was then stopped, and the solution was continuously shaken slowly until it cooled.

### Preparation of 1F12-conjugated AuNP and magnetic nanoparticles

AuNP were synthesized using the citrate reduction as per the HAuCl_4_ protocol [52]. First, 1F12 antibody was purified with protein A immunoaffinity column, and conjugated to AuNP according to the following steps: (1) 1 mL of AuNP was added to a 1.5 mL centrifuge tube, followed by the addition of 16 μL of 0.1 M K_2_CO_3_ with thorough mixing; (2) 25 μg of 1F12 was added to the mixture for 15 min, keeping it undisturbed for another 15 min at 25°C; (3) 10 μL of 10% BSA was added to the mixture with shaking for 15 min and the mixture was then kept undisturbed for 15 min at 25°C to achieve a sealing effect on the surface of AuNP; (4) the mixture was centrifuged at 13,500 rpm for 10 min, and the precipitate was collected to resuspend in 1 mL of PBS (0.01 M, pH 7.4, including 0.03% Tris, 2% Sucrose, 0.2% Casein, 1% BSA, 0.1% PVP, 1% NaN_3_, 0.1% PEG, 0.05% Tween-20) and stored at 4°C for further use.

Further, 1F12-conjugated magnetic nanoparticles (1F12-MNPs) were prepared by making the amino group of 1F12 react with the carboxyl group of MNPs, as we previously described [53]. Briefly, 1 mL MNPs (10 mg) was washed twice with 1 mL deionized water followed to wash with 4-Morpholineethanesulfonic acid (MES) buffer (0.02 M MES, pH5.0). Then, the MNPs were resuspended in 200 μL of MES buffer and then 300 μg of 1F12 (200 μL) was added for 30 min reaction at 25°C in shaking. Next, 100 μL of freshly prepared EDC-HCl buffer (52.2 mM, dissolved in 0.02 M MES, pH5.0) was added to react in shaking overnight, followed to be blocked with blocking buffer (1% BSA dissolved in 0.02 M MES, pH5.0). Finally, the prepared 1F12-MNPs were stored in the storage buffer (PBS containing 0.1% Tween-20 and 0.02% NaN_3_, pH 7.4) at 4°C for further use. The prepared antibody-modified MNPs were evaluated via reduced tris-tricine SDS–polyacrylamide gel electrophoresis (SDS-PAGE), ELISA, and immunoprecipitation followed with Western blotting (IP-Western blotting).

### Characterization of AuNP, MNP, Aβ_42_Ms, and Aβ_42_Os

The prepared AuNP, magnetic nanoparticle (MNP), Aβ_42_Ms, and Aβ_42_Os were confirmed by transmission electron microscopy (TEM) and scanning electron microscopy (SEM), respectively. In short, a small drop of samples (5 μL) was deposited onto a copper grid, and the excess liquid was removed by blotting using a filter paper, thus leaving a thin film of the solution on the grid. Subsequently, Tecnai G20 transmission electron microscope (FEI Ltd., USA) and Phenom Pharos scanning electron microscope (SEM) (Phenom Ltd., NLD) were used to characterize the morphology of the abovementioned samples. The particle size distribution and Zeta-potential of all nanoparticles were measured via dynamic light scattering (photon correlation spectroscopy) on a Zetasizer Nano-ZS90 system (Malvern Instruments, Worcestershire, UK).

### Immunoprecipitation and Western blotting

The prepared Aβ_42_ samples, including Aβ_42_Ms and Aβ_42_Os, were incubated with 1F12-MNPs for 30 min at 25°C, and then the enriched Aβ_42_ was eluted with 0.1 M glycine (pH 3.0) and denatured in loading buffer (Boster Biotech, USA) for 10 min at 95°C, followed to run on a 12% reducing SDS-PAGE. The proteins were transferred onto a polyvinylidene fluoride membrane at 160 mA for 1 h. The membrane was blocked with 5% skimmed milk dissolved in PBS-T buffer, and then incubated with the primary antibody (1F12 or 2C6) and the secondary antibody HRP-conjugated goat anti-mouse IgG (H + L) in an order. The membrane was washed three times with PBS-T buffer (KCl 2.7 mM, KH_2_PO_4_ 2 mM, NaCl 137 mM, Na_2_HPO_4_ 10 mM, 0.05% Tween-20, pH 7.4) and the immunological signals were detected using the ECL-substrate (Vazyme, China) on Tanon 5200 Muiti (Shanghai, China).

### ELISA

For indirect ELISA, the wells of a 96-well plate (Corning Inc., USA) were coated with 0.5 μg/well of Aβ_42_ overnight at 4°C. The plate was blocked with 5% skimmed milk, and then incubated with primary antibodies (1F12, AuNP-1F12, or 1F12-MNPs) and the secondary antibody HRP-conjugated goat anti-mouse IgG (H + L) in an order. The 96-well plate was washed four times with PBS-T in each step. The immunoreaction was visualized by TMB substrate solution (Tiangen Biotech, Beijing, China) and detected with an Epoch Microplate Spectrophotometer (Bio Tek, USA) at 450 nm.

For competitive ELISA, 96-well plates were coated with 0.5 μg Aβ_42_ in each well and blocked with 5% skimmed milk. Triplicates of biotinylated 1F12 or 2C6 (250 ng/mL) were mixed with serially diluted Aβ preparations of Aβ_40_, Aβ_42_Ms, and Aβ_42_Os diluted in PBS with the final concentration from 25 μM to 5 pM. After 1 h pre-incubation at 4°C in 1.5 mL tubes, the antibody-antigen mixtures were incubated on the Aβ_42_ antigen-coated plates for 1 h at 25°C. After incubating with streptavidin-coupled poly-HRP, the immunoreaction was visualized and detected as above described.

### Immunofluorescence assay

The 15-μm coronal frozen sections of the brain tissue samples were permeabilized with 0.2% Triton X-100 for 20 min at 25°C, then blocked with 3% bovine serum albumin for 2 h, followed to incubate with Cy3-conjugated mAb 1F12, 2C6 and Iba 1 for overnight at 4°C. Besides, the slides were counterstained with thioflavin S and 6-diamino-2-phenylindole (DAPI; Thermo Fisher Scientific). The fluorescence signals were detected by Zeiss LSM710 confocal microscope.

### Optimization of key parameters

To keep the dLFI with the optical working condition, we detail to optimize the volume of K_2_CO_3_ and the mAb 1F12 concentration of conjugation. For the volume of K_2_CO_3_ optimization, the 1 mL of the AuNP solution was adjusted to 4, 8, 12, 16, 20, 24, 28, and 32 μL of 0.1 M K_2_CO_3_. While for mAb 1F12 concentration optimization, the mAb 1F12 was added dropwise to 1 mL of AuNP solution at final concentrations of 5, 10, 15, 20, 25, and 30 μg/mL.

### Preparation of dLFI

**Scheme 1:**
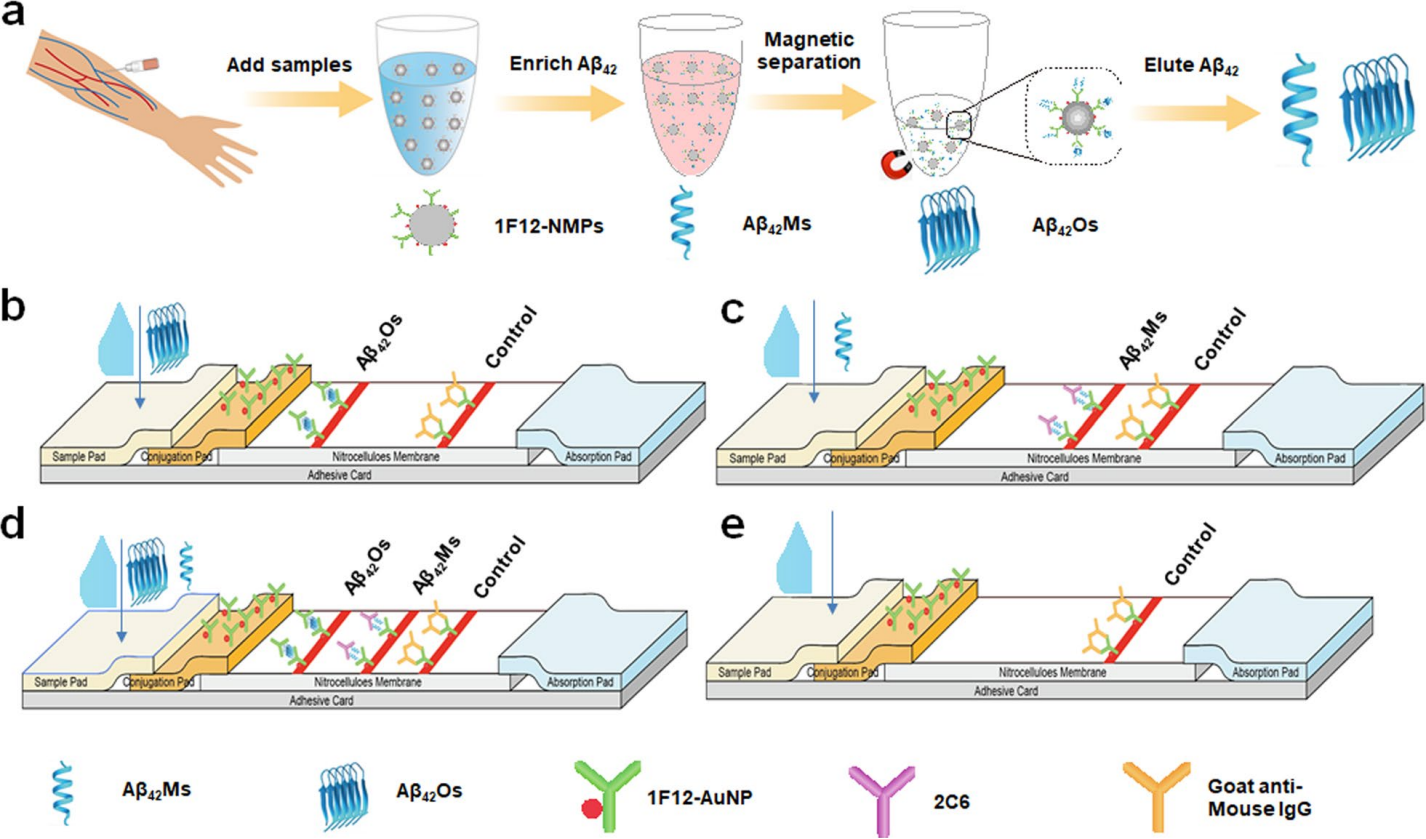
The principle and test procedure of dLFI. **a** The blood samples were enriched by the mAb 1F12-modified MNPs and then eluted for dLFI analysis. Schematic representation of the working principle of the dLFI for the detection of Aβ_42_Os (**b**), Aβ_42_Ms (**c**), Aβ_42_Ms and Aβ_42_Os (**d**), or without Aβ_42_Ms or/and Aβ_42_Os (**e**)

Scheme 1b describes the main components of a one-piece dLFI, which mainly includes sample pads, conjugation pads, signal pads, and absorbent pads assembled on a plastic backplane in sequence. The sample pad was impregnated with a buffer solution (0.01 M PBS, pH 7.4, including 1% BSA, 2% Sucrose, 0.1% PEG, and 0.05% Tween-20) and air-dried overnight before use. The goat anti-mouse IgG antibody, 1F12, and 2C6 were coated on the NC membrane to form a control line and two test lines. The coated NC membrane was dried for 30 min and blocked with 1% (w/v) BSA dissolved in 0.01 M PBS for 2 h at 25°C. After blocking, the membrane was washed three times with PBS containing 0.05% [v/v] Tween-20 (PBS-T) and dried at 25°C. Following this, the dLFI was assembled in the following order: the sample pad was first placed on the PVC plate, overlapping the anti-Aβ_42_ mAb 1F12 conjugate pad by 2 mm, and the bottom of the antibody-coated NC membrane was then overlapped by 2 mm. Next, the absorbent pad was overlapped on the top of the NC membrane by 2 mm. After assembly, the plate was evenly cut into 3.5 mm wide strips, sealed with a desiccant, and stored at 25°C. The concentration of 1F12 coated on the first test line and 2C6 coated on the second test line was 1 mg/mL. The concentration of goat anti-mouse IgG antibody coated on the control line was 0.5 mg/mL.

### Detection principle and test procedure of dLFI

Our multicomponent biosensor system developed for rapidly and simultaneously detecting Aβ_42_Ms and Aβ_42_Os was based on two groups of paired mAbs. Due to Aβ_42_Os was aggregated by several Aβ_42_Ms, we used the group of 1F12/1F12 with the same epitope that ensures only Aβ_42_Os was detected but not Aβ_42_Ms, which need a pair of antibodies with different epitopes just liking our described as the group of 1F12/2C6. The following steps were included in dLFI assay process: the antibody-modified MNPs were used for the enrichment of Aβ peptides dissolved in the samples and then eluted with 0.1 M glycine (pH 3.0), followed by immediate neutralization to pH 7.4 using a neutralization buffer (1 M Tris–HCl, pH 8.5). Each collected solution (approximately 50 μL) was dripped onto the sample pad. Aβ_42_Ms and/or Aβ_42_Os in samples were first recognized by 1F12–AuNP conjugate mixture on the conjugate pad and migrated along the NC membrane via capillary action. The Aβ_42_Os–1F12–AuNP complex was first solubilized by 1F12 coated on the NC membrane to form the test line 1, whereas Aβ_42_Ms–1F12–AuNP complex continued to migrate until it was solubilized by mAb 2C6 coated on the NC membrane to form the test line 2. The remaining 1F12–AuNP were captured by goat anti-mouse IgG coated on the NC membrane to form the control line. The test results were evaluated within 5 min.

### Preparations of oligomeric and monomeric Aβ

The detailed steps of oligomeric and monomeric Aβ preparations were described in our previous report [51]. In brief, Aβ_42_Ms and Aβ_40_Ms were obtained by dissolving lyophilized Aβ_42_ or Aβ_40_ peptides in 1,1,1,3,3,3-hexafluoroisopropanol (HFIP) for overnight incubation at 25°C. HFIP was evaporated with nitrogen to form a thin film, and Aβ was re-dissolved in dimethyl sulfoxide. Aβ_42_ oligomers (Aβ_42_Os) and Aβ_40_ oligomers (Aβ_40_Os) were obtained from 50 μM Aβ_42_ or Aβ_40_ monomer solution after 24 h incubation at 37°C in the dark, respectively.

### Specificity and sensitivity of the dLFI

Several different compounds such as Aβ_40_Ms, Aβ_40_Os, p-Tau^396,404^, Cis-Tau, p-Tau^231^, and BSA were detected to evaluate the specificity of the dLFI. For sensitivity evaluation, a series of synthetic Aβ_42_Ms and Aβ_42_Os dilutions, ranging from 625 ng/mL to 154 pg/mL, were prepared by diluting with PBS. Each dilution was applied to the dLFI test, and the detection limit was determined. The visual limit of detection is defined as the minimum concentration of Aβ_42_Ms and Aβ_42_Os that leads to the complete disappearance of the test line.

### Analysis of blood samples using dLFI

All procedures involving animal studies have been reviewed and approved by the Institutional Animal Care and Use Committee of Huazhong University of Science and Technology. Human blood samples were obtained from the patients/participants in Union Hospital of Huazhong University of Science and Technology who have been provided with their written informed consent to participate in this study.

The blood samples of 5xFAD mice (Stock No: 34848-JAX) aged 3 months (n = 4) and 9 months (n = 4) were collected and used to evaluate the analytical performance and applicability of the dLFI. A mixture of Aβ_42_Ms and Aβ_42_Os was used as a positive control, while blood samples from C57BL/6 J mice at 3 months (n = 3) and 9 months old (n = 3) were used as negative controls. In addition, blood samples collected from HC (n = 7) and AD patients (n = 8) were analyzed with dLFI. The detailed information of the participant in this manuscript is summarized in Additional file 1: Table S1.

### Validation and analysis with sandwich ELISA

The blood samples of 5xFAD or C57BL/6 J mice at 3 months (n = 4) and 9 months of age (n = 4), and HC (n = 7) and AD patients (n = 8) were analyzed via a prepared sandwich ELISA for Aβ_42_ monomers and Aβ_42_ oligomers as our previous described [51].

### Statistical analyses

The data are presented as means ± SD. Unpaired *t*-test was used for two-group comparisons. One-way analysis of variance (ANOVA) was used for multigroup comparisons. Statistical significance is represented in the figure by *p < 0.05, **p < 0.01, ***p < 0.001, ****p < 0.0001, and n.s. (indicating no significance). All statistical analyses were performed with GraphPad Prism 8.0 software.

## Results and discussion

### Mechanism of the proposed biosensor

In this study, we combined the biomarker measurement of Aβ_42_Ms and Aβ_42_Os to obtain a more accurate diagnosis of AD. To further verify the results, we developed dLFI using two matched antibody pairs 1F12/1F12 and 1F12/2C6 to obtain a rapid on-site response and accurate detection tests and to determine the changes of Aβ_42_Ms and Aβ_42_Os in the blood for clinical validation of AD diagnosis. As shown in Scheme 1a, the blood samples were firstly enriched with MNPs modified by mAb 1F12, and then the Aβ_42_Ms or/and Aβ_42_Os were eluted for LFI analysis. The eluted Aβ_42_Ms or Aβ_42_Os were added to the sample pad and then recognized by the 1F12–AuNP conjugate mixture on the conjugate pad and migrated along the NC membrane via capillary action. The Aβ_42_Os–1F12–AuNP complex was first fixed by 1F12 coated on the NC membrane to form the test line 1, due to Aβ_42_Os were aggregated by multiple monomers, exposing several same epitopes that could be recognized by the same detection antibody (Scheme 1b). This strategy is only used for the detection of Aβ_42_Os. The Aβ_42_Ms–1F12–AuNP complex continued to migrate and was solubilized by mAb 2C6 with different epitopes toward Aβ_42_ (specific to amino acids 3–9, 13–19, 18–25, 29–36, and 36–42 of Aβ_42_ sequence). Compared to 1F12 (specific to amino acids 3–9), Aβ_42_Ms were only detected by a pair of antibodies with different epitopes (Scheme 1c). If Aβ_42_Ms and Aβ_42_Os are present in the sample, the 1F12–AuNP conjugates will be combined with the two test lines and the control line (Scheme 1d). If there is no Aβ_42_ in the sample solution, the 1F12–AuNP conjugates will not bind to the two test lines but will show the control line, forming a red band (Scheme 1e).

### Characterization of mAb 1F12 and 2C6

**Fig. 1:**
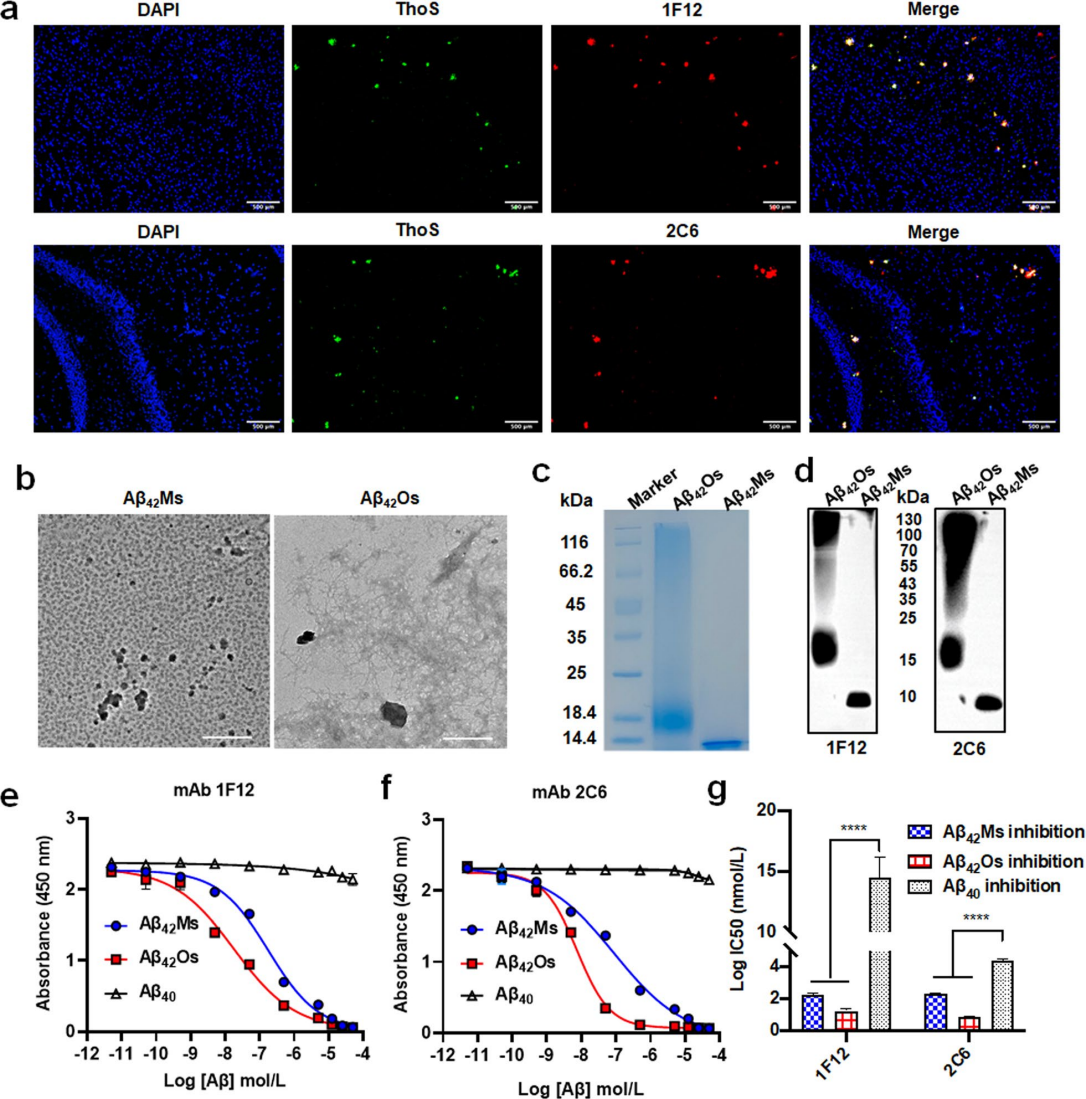
Characterization of conformation-specific antibodies for Aβ_42_Ms and Aβ_42_Os. **a** Confocal fluorescence images of mouse 5xFAD brain sections using Cy3-labeled anti-Aβ_42_ monoclonal antibody 1F12 or 2C6 and thioflavin S. (Scale bar: 500 μm). **b** Parallel analysis of the morphology of Aβ_42_Ms and Aβ_42_Os by cryo-transmission electron microscopy. (Scale bar: 500 nm). The SDS-PAGE (**c**) and Western blotting (**d**) analysis of the prepared Aβ_42_Ms and Aβ_42_Os using 1F12 or 2C6. The binding selectivity of 1F12 (**e**) and 2C6 (**f**) to Aβ_42_ and Aβ_40_ was determined by competitive ELISA. **g** The logIC50 value of 1F12 or 2C6 to Aβ_42_ and Aβ_40_. The data are presented as means ± SD, n = 3 in **e** and **f**. One-way analysis of variance (ANOVA) was used for multigroup comparisons. Statistical significance is indicated in the figures by ****p < 0.0001

We first evaluated whether the two antibodies could stain Aβ plaques in the brains of 5xFAD mice. The results of confocal fluorescence images showed that Aβ plaques were stained by Cy3-labeled 1F12 or 2C6 and co-localized with thioflavin S, and its functional characteristic was to bind Aβ plaques (Fig. 1a) [54–56]*.* The binding selectivities of 1F12 and 2C6 for different Aβ species were detected by competitive ELISA. The morphology of the prepared Aβ_42_Ms and Aβ_42_Os was first confirmed by TEM. The results showed that the morphology of Aβ_42_Ms was coil structures (Fig. 1b, left), while Aβ_42_Os presented a β-sheet and typical fibril structure (Fig. 1b, right). A 12% reduced SDS-PAGE gel was used to confirm the molecular weight and components of the prepared Aβ_42_Ms and Aβ_42_Os (Fig. 1c). Western blot results showed that both Aβ_42_Ms and Aβ_42_Os were well recognized by 1F12 or 2C6 (Fig. 1d). Competitive ELISA was used to evaluate the binding selectivity of 1F12 and 2C6 to different Aβ species. Figures 1e and f show that 1F12 and 2C6 have high binding selectivity to Aβ_42_ species rather than Aβ_40_. The IC50 values of 1F12 for Aβ_42_Ms and Aβ_42_Os were 180.8 nM and 18.1 nM, respectively. While for 2C6, the IC50 values for Aβ_42_Ms and Aβ_42_Os were 90.3 nM and 7.5 nM, respectively (Fig. 1g). The IC50 values plus the K_d_ values of 1F12 (K_d_ = 1.66 ± 0.09 nM for Aβ_42_Ms and K_d_ = 0.38 ± 0.04 nM for Aβ_42_Os) and 2C6 (K_d_ = 3.59 ± 0.27 nM for Aβ_42_Ms and K_d_ = 0.61 ± 0.03 nM for Aβ_42_Os) that were reported in our previous study indicated [51] that 1F12 and 2C6 have high binding affinity and selectivity for both Aβ_42_Ms and Aβ_42_Os.

### Characterization of AuNP and AuNP-1F12 conjugates

**Fig. 2:**
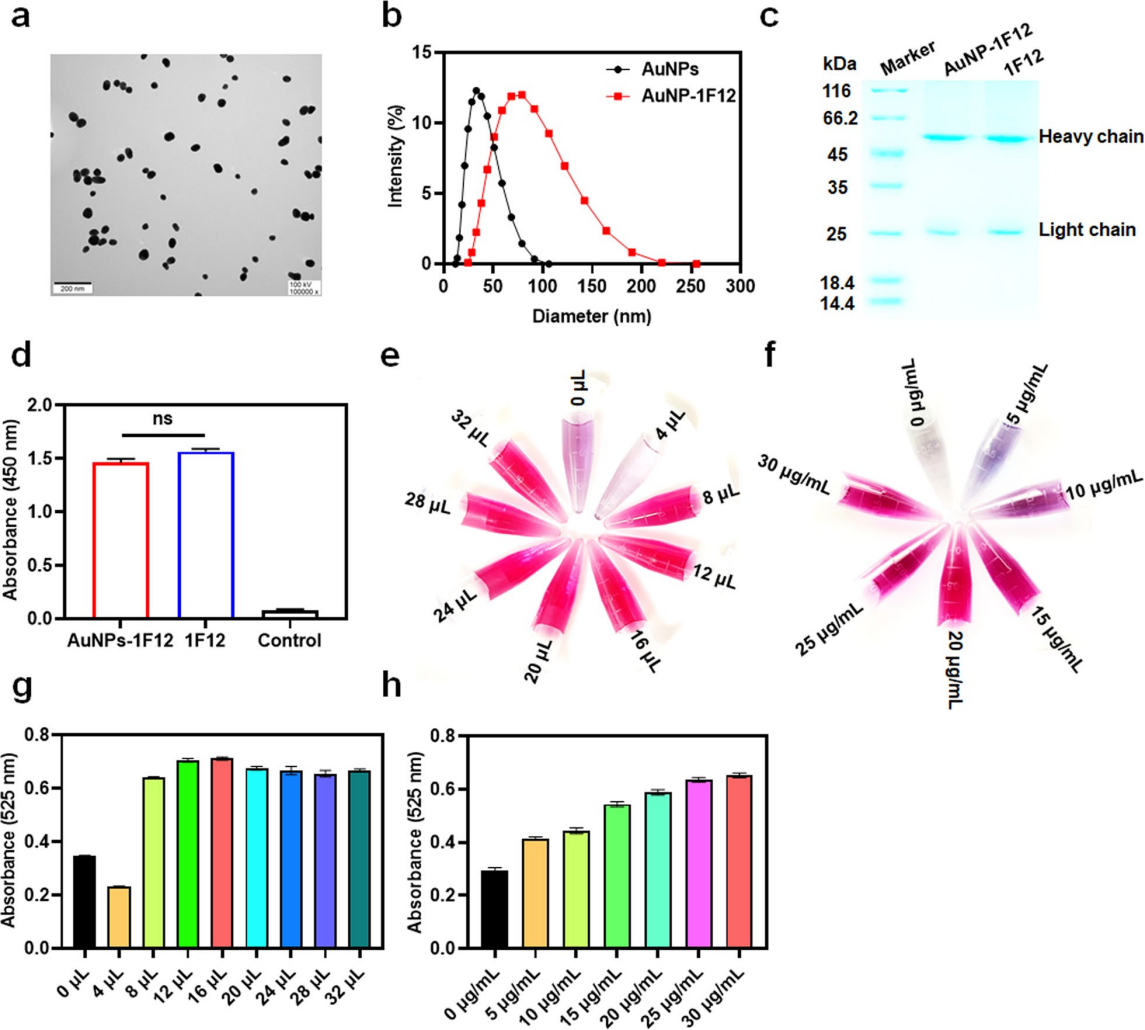
Characterization of AuNP–1F12 conjugates. **a** TEM images of synthetic AuNP. (Scale bar: 200 nm). **b** The size distributions of AuNP before and after modification. **c** A 12% reduced SDS-PAGE gel analysis of AuNP–1F12 and 1F12. **d** ELISA was used to determine the bioactivity of AuNP–1F12 conjugates. The optimal volume of K_2_CO_3_ for the conjugation of 1F12 with AuNP was evaluated by the color (**e**) and OD_525_ value (**g**). The color (**f**) and OD_525_ value (**h**) were used to evaluate the optimal concentration of 1F12 of synthetic AuNP-1F12. For Fig. 3b, d, g, and h**,** the data are presented as mean ± SD, n = 3. Statistical significance is indicated in the figures by n.s. (indicating no significance)

The synthesized AuNP had good dispersion and uniformity with wine red, and a characteristic single absorption peak was found at 525 nm (Additional file 1: Fig. S4a). The morphology and size of prepared AuNP were characterized using a Tecnai G20 transmission electron microscope and dynamic light scattering with an average size of 40 nm (FEI Ltd., USA) (Fig. 2a and b). After being conjugated with 1F12, the size distribution and ζ-potential of AuNP–1F12 increased significantly with the size from 40 nm (before conjugation) to approximately 80 nm (after conjugation) (Fig. 2b) and ζ-potential from − 38 (before conjugation) to approximately − 23 (after conjugation) (Additional file 1: Fig. S4b). Besides, the absorption spectrum of 1F12 was measured, and the results showed a sharp decrease in the absorption peak at 280 nm after conjugation with AuNP (Additional file 1: Fig. S4c). Coupled with the results of the 12% reduction SDS-PAGE gel, they jointly confirmed the successful combination of 1F12 to AuNP (Fig. 2c). The ELISA was used to evaluate the bioactivity of AuNP-1F12 conjugate and results showed that both 1F12 and AuNP-1F12 reacted well with Aβ_42_ (Fig. 2d).

### Optimization of the dLFI

To improve the sensitivity, accuracy, and reproducibility of the dLFI test, the capture antibody 1F12–AuNP was optimized during the preparation process. We first optimized the volume of K_2_CO_3_, which is a commonly used buffer to adjust the pH of AuNPs solution to better bind the antibody. Figure 2e and g show the effect of K_2_CO_3_ volume on the 1F12–AuNP conjugates. As the volume of K_2_CO_3_ increased from 4 to 16 μL, the color, optical density (OD) value, and absorption spectrum at 525 nm of the 1F12–AuNP solution gradually increased, the color of the solution changed from lavender to wine red (Fig. 2e), and the OD_525_ value increased from 0.23 to 0.71 (Fig. 2g). With a further increase from 20 to 32 μL, the OD_525_ value (Fig. 2g) and the absorption spectrum (Fig. S4d) decreased slightly. To obtain a higher colorimetric ratio, 16 μL was selected as the optimal volume of K_2_CO_3_ of antibody coupling, and the pH value of the AuNPs solution was approximately 8.5. When the pH value is equal to or slightly high than the isoelectric point (pI) of the antibody (pI = 8), the antibody is electrically neutral, resulting in a small electrostatic interaction between the antibody and AuNPs, so that the antibody is more easily to adsorb on the surface of AuNPs [57–59]. When the pH value is less than the pI, the antibody is positively charged. Since the AuNP is negatively charged, the antibody is easily adsorbed to form large polymers, leading to the aggregation of AuNPs [57–59]. On the contrary, once the pH is higher than the pI, the antibody will be negatively charged and repel the negatively charged AuNPs, causing them to fail to bind to each other [57–59].

As shown in Fig. 2f and h, as the concentration of 1F12 increases from 5 to 25 μg/mL, the color gradually changes from purple to wine red (Fig. 2f), and the OD_525_ value (Fig. 2h) increases from 0.41 to 0.64. However, when the concentration of 1F12 was as high as 30 μg/mL, the OD_525_ value (Fig. 2h) and absorption spectrum (Additional file 1: Fig. S4e) only slightly increased. Therefore, the optimal concentration of 1F12 was 25 μg/mL. The significant change in the color of the AuNP solution during the antibody coupling process could be explained by the fact that the AuNPs cannot be fully labeled when no antibody is added or the amount of added antibody is insufficient. The unlabeled AuNPs were aggregated under the action of 10% NaCl. On the contrary, only when the amount of antibody reaches or slightly exceeds the stable amount, the AuNPs can remain stable [60, 61].

### Specificity and sensitivity of the dLFI

**Fig. 3:**
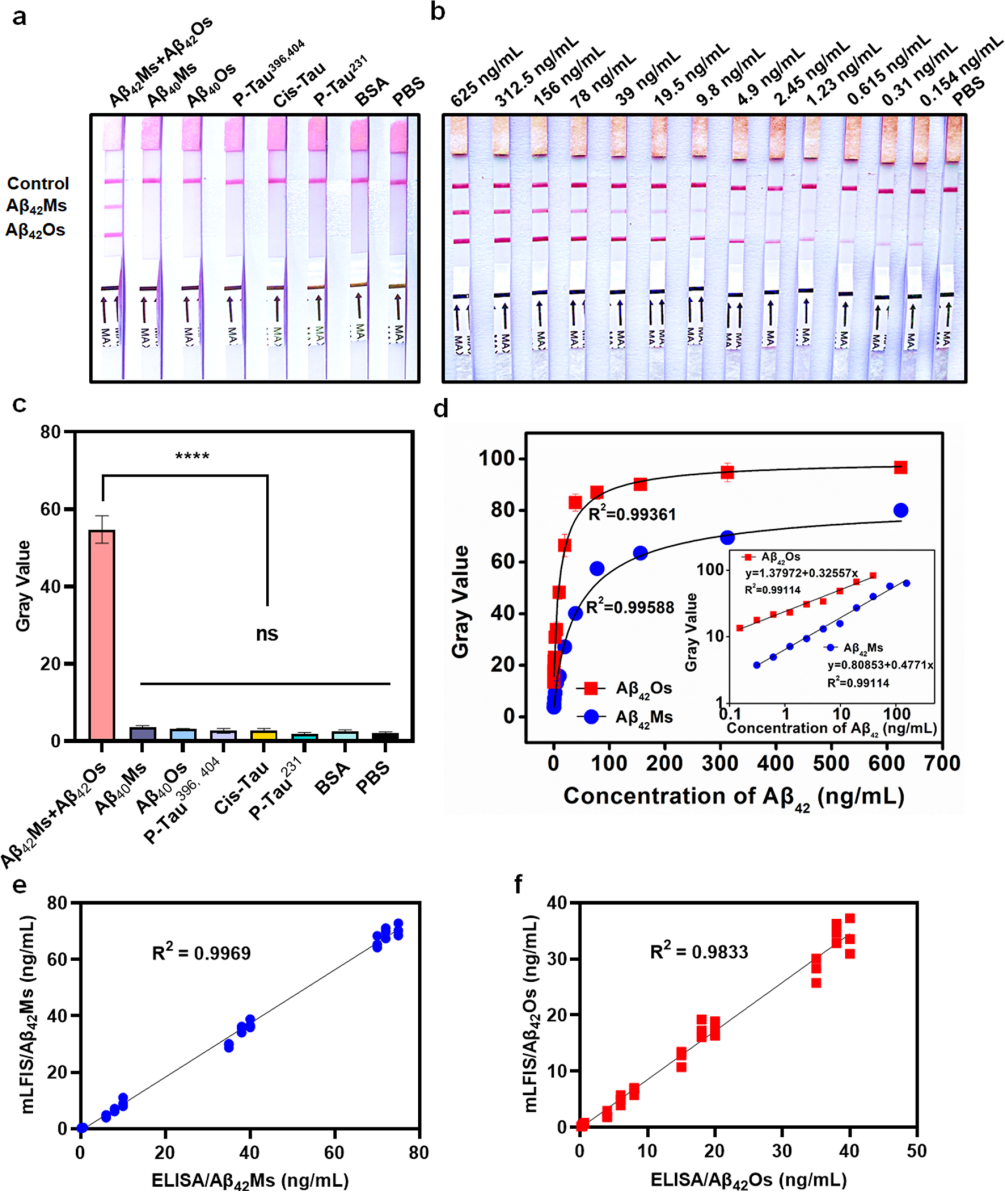
The performance of the dLFI. The visual result (**a**) and gray value (**c**) on the T line of the dLFI in the specificity assay, n = 3. **b** The sensitivity of dLFI for the simultaneous detection of Aβ_42_Ms and Aβ_42_Os. **d** The plotted linear curve of different Aβ_42_ conformations, n = 3. **e**, **f** Correlation between results from dLFI (Y-axis) and sandwich ELISA (X-axis) in spiked samples, n = 12. Data are presented as means ± SD. One-way analysis of variance (ANOVA) was used for multigroup comparisons. Statistical significance is represented in the figures by ****p < 0.0001 and n.s. (indicating no significance)

The specificity of dLFI was evaluated among six compounds. As shown in Fig. 3a, dLFI can detect a mixture of Aβ_42_Ms and Aβ_42_Os, and does not cross-react with the tested peptides, indicating that dLFI based on 1F12/1F12 and 1F12/2C6 antibody pairs could accurately detect both Aβ_42_Os and Aβ_42_Ms (Fig. 3a and c). Therefore, dLFI can be used to effectively evaluate multiple analytes. Each test line in the strip has a cut-off value, which is the minimum Aβ_42_Ms or Aβ_42_Os concentration required to make the test line visible in samples. For Aβ_42_Ms or Aβ_42_Os, the cut-off value of the dLFI was 154 pg/mL (Fig. 3b). Different concentrations of Aβ_42_Ms and Aβ_42_Os were used in dLFI to generate two standard curves. The color intensity of the test line is directly proportional to the concentration of the analyte in samples. Figure 3d shows two standard curves of dLFI. The linear relationship of dLFI is y = 1.37972 + 0.32557x, and the LOD of Aβ_42_Os is 154 pg/mL (Fig. 3d, inserted Figure, red square). The linear relationship of dLFI is y = 0.80853 + 0.4771 × with LOD of 154 pg/mL for Aβ_42_Ms (Fig. 3d, inserted Figure, blue circle).

### The reliability and practicability of the proposed dLFI

The reliability and practicability of the proposed dLFI sensor in spiked samples were verified and compared by sandwich ELISA. The spiked concentration conformed to the linear range. Therefore, the results (Fig. 3e and f) showed that the two methods were well consistent. The correlation coefficients of Aβ_42_Ms and Aβ_42_Os were 0.9969 and 0.9833, respectively. Although the sensitivity of dLFI is relatively low, the dLFI sensor requires less than 30 min of enrichment and immunoreaction time to complete sample analysis, while traditional sandwich ELISA requires about 3 h.

### The performance of the dLFI in detecting Aβ_42_Os and Aβ_42_Ms in 5xFAD mice

**Fig. 4:**
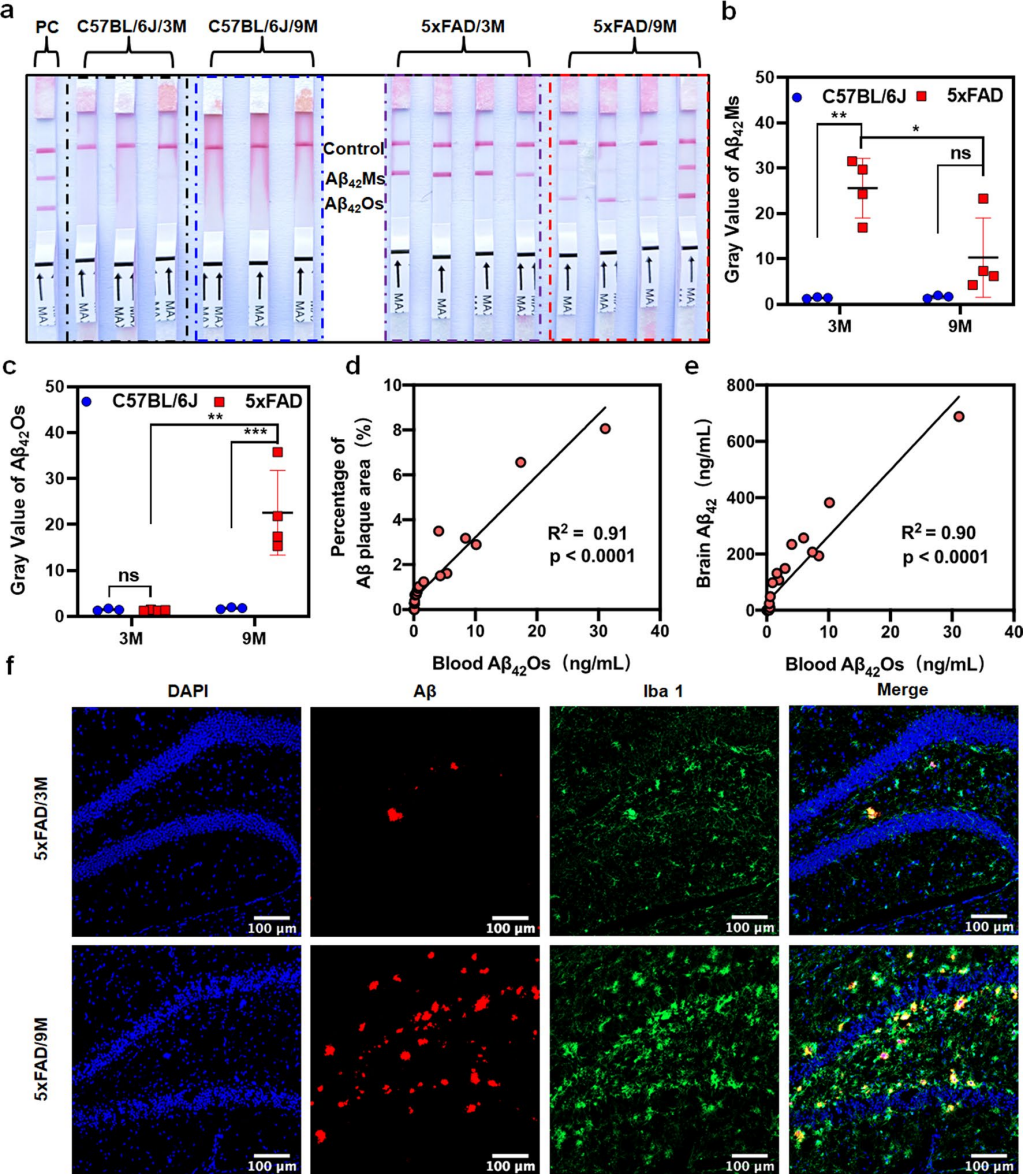
Simultaneous detection of Aβ_42_Ms and Aβ_42_Os in the blood of 3- and 9-month-old 5xFAD and C57BL/6 J mice. The visual results (**a**) and grey value (**b**, **c**) on the T-line of the dLFI in blood samples from 5xFAD (n = 8) and C57BL/6 J (n = 6) mice at 3 and 9 months old. **d**, **e** Correlation between blood Aβ_42_Os level and Aβ plaque area (**d**, p < 0.0001) or soluble Aβ_42_ level (**e**, p < 0.0001) in the brain of 5xFAD mice, a total of n = 25, of which n = 5 in each age group, including 1, 3, 6, 9, and 12 months old. **f** Confocal fluorescence images of Aβ plaques and Iba 1-positive cell staining in 3 or 9-month-old 5xFAD mice. (Scale bar: 100 μm). Data are presented as means ± SD. Unpaired *t*-test was used for two-group comparisons. Statistical significance is indicated in the figures by **p < 0.01, ***p < 0.001 and n.s. (indicating no significance)

To check the performance of the dLFI in the actual tests, blood samples of 5xFAD (AD model mice) or C57BL/6 J mice (control mice) at 3 and 9 months old were collected for dLFI detection. The mixture of Aβ_42_Ms and Aβ_42_Os was used as a positive control. The results showed that in the blood of C57BL/6 J mice at 3 and 9 months old, dLFI could hardly detect Aβ_42_Ms or Aβ_42_Os (Fig. 4a, left, black and blue box, and Fig. 4b-c). Interestingly, the dLFI results showed that Aβ_42_Ms were the main form of Aβ_42_ in 3-month-old 5xFAD mice (Fig. 4a, right, purple box, and Fig. 4b), but the results were different in 9-month-old 5xFAD mice. In 9-month-old 5xFAD mice, the color of Aβ_42_Os in the first test line was significantly enhanced, but Aβ_42_Ms were hardly observed in three of the four 5xFAD mice (Fig. 4a, right, red box, and Fig. 4c). Only one of the four 9-month-old 5xFAD mice showed high levels of Aβ_42_Ms and Aβ_42_Os. These results revealed that Aβ_42_Ms appeared in the peripheral blood of 5xFAD mice at an early stage [e.g., 3-month-old 5xFAD mice had less Aβ plaque load and Iba 1-positive cells staining (Fig. 4f, up)]; as the disease progresses, high levels of Aβ_42_Os and insoluble Aβ plaques were the main forms in the brain. As Aβ_42_Ms in the brain gradually aggregated into Aβ_42_Os, soluble Aβ_42_ could enter the blood, resulting in a decrease in Aβ_42_Ms level and an increase in Aβ_42_Os level [e.g., 9-month-old 5xFAD mice had more Aβ plaque load and Iba 1-positive cells staining (Fig. 4f, down)]. Of note, this elevated Aβ_42_Os level was closely related to the Aβ plaque area (Fig. 4d, p < 0.0001) and soluble Aβ_42_ level (Fig. 4e, p < 0.0001) in the brain of 5xFAD mice, indicating that its level may reflect the progress of the disease. The phenomenon observed in 5xFAD mice was that the accumulation of Aβ_42_Os is directly accompanied by the decrease of Aβ_42_Ms, consistent with the typical clinical symptoms of AD patients [62–67]. Altogether, our results showed that Aβ_42_Os or Aβ_42_Ms that are mis-detected by commonly used ELISA are valuable biomarkers for AD diagnosis when they are accurately distinguished and detected.

### The performance of the dLFI in detecting Aβ_42_Os and Aβ_42_Ms in AD patients

**Fig. 5:**
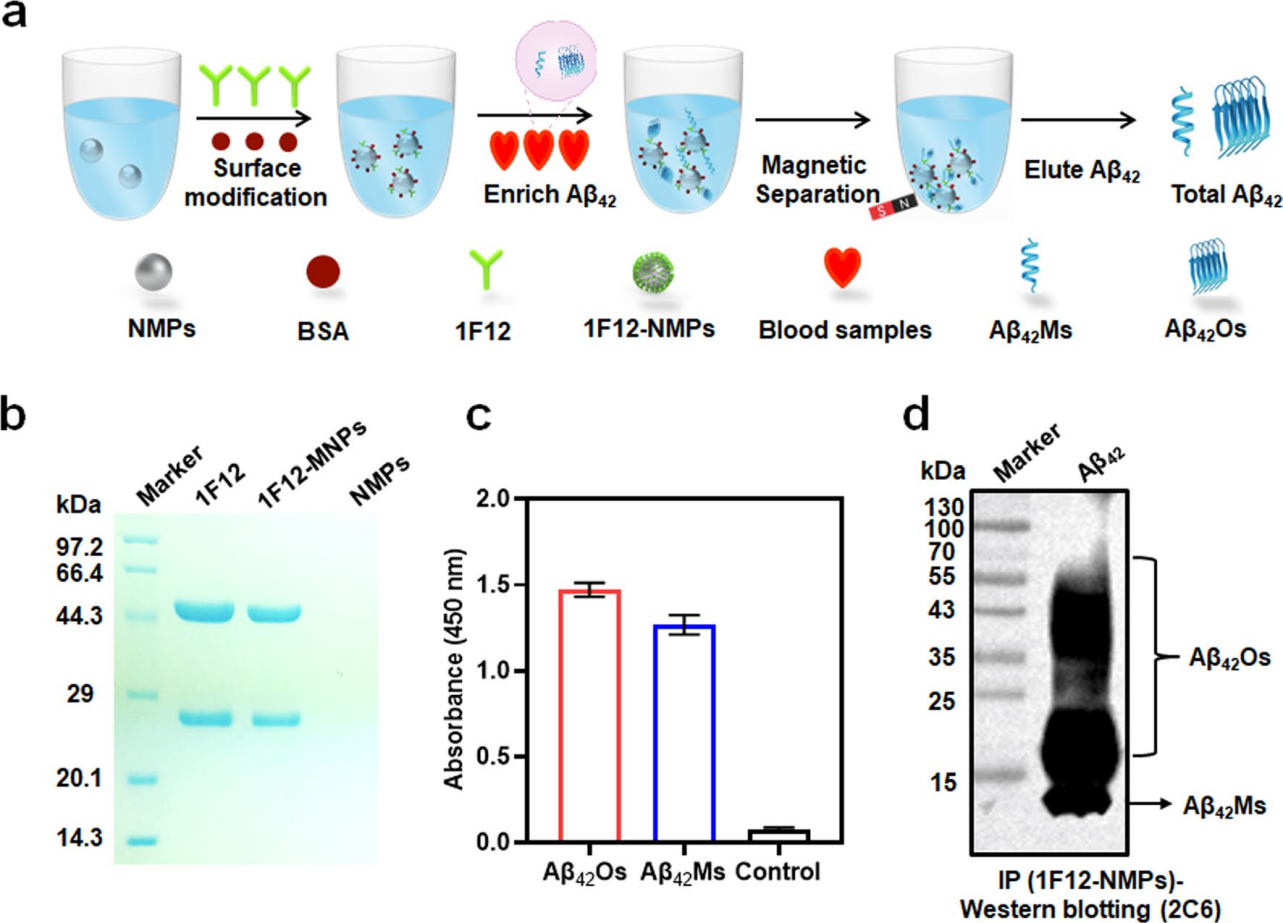
Characterization of antibody-modified NHS-magnetic nanoparticles (MNPs). **a** Schematic illustration of the working steps for the use of antibody-modified MNPs for the enrichment of Aβ_42_Os and/or Aβ_42_Ms. **b** A 12% reduced SDS-PAGE gel analysis of 1F12-MNPs. 1F12 and NMNs were used as controls. ELISA (**c**) and IP-Western blotting (**d**) analysis for the bioactivity of 1F12-MNPs. Data are presented as means ± SD, n = 3 in **c**

**Fig. 6:**
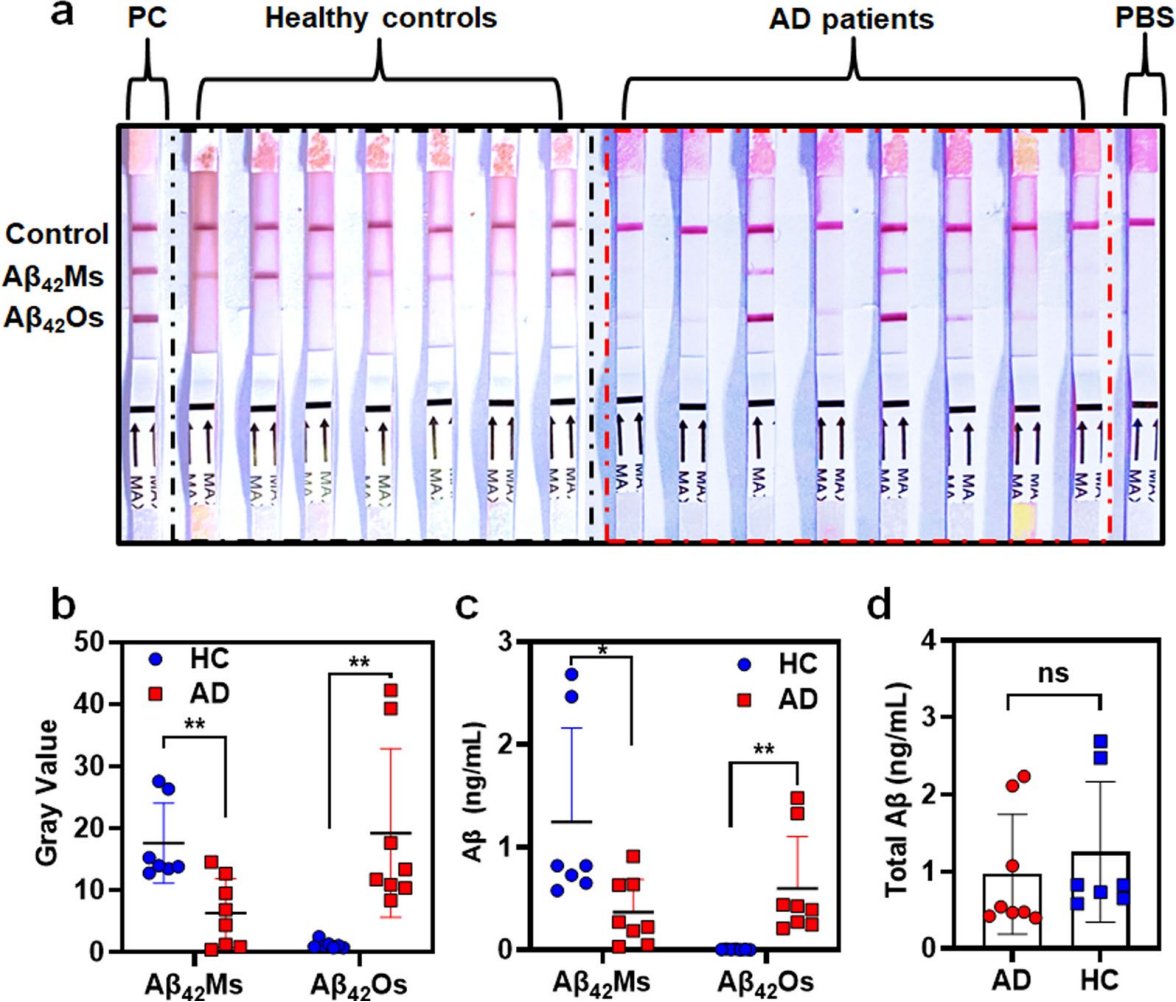
Detection of Aβ_42_Ms and/or Aβ_42_Os in human blood samples by dLFI. The visual results (**a**) and grey value (**b**) of the Aβ_42_Ms and/or Aβ_42_Os level in the blood of AD patients (n = 8) or healthy controls (n = 7) were analyzed by dLFI. **c** The Aβ_42_Ms and/or Aβ_42_Os levels in the blood of AD patients (n = 8) or healthy controls (n = 7) were detected by sandwich ELISA. **d** Total Aβ_42_ levels in human blood samples were detected by sandwich ELISA (n = 8 for AD patients and n = 7 for healthy controls). Data are presented as means ± SD. Unpaired *t*-test was used for two-group comparisons. Statistical significance is indicated in the figures by *p < 0.05, **p < 0.01 and n.s. (indicating no significance)

To further improve the sensitivity and accuracy of the dLFI in the analysis of human blood samples, immunocapture magnetic beads were used to enrich the Aβ_42_Os and Aβ_42_Ms in samples (Fig. 5a). The unmodified MNPs were characterized and found to be approximately 190 nm (Additional file 1: Fig. S5a). After conjugation with 1F12, compared with bared MNPs, the size and Zeta potential of MNPs showed significant changes (Additional file 1: Fig. S5b–d). Furthermore, a 12% reduced SDS-PAGE gel showed two typical bonds, including the light and heavy chains observed in the lanes of 1F12 and 1F12-MNPs, confirming the successful conjugation of 1F12 with MNPs (Fig. 5b). The biological activity of 1F12-MNPs was assessed by ELISA and IP-Western blotting. The results of ELISA (Fig. 5c) and IP-Western blotting (Fig. 5d) confirmed that 1F12-MNPs inherited the binding affinity of 1F12 for Aβ_42_Ms and Aβ_42_Os. After using dLFI to detect blood samples of HC (n = 7) and AD patients (n = 8), the results showed that the Aβ_42_Ms level of HC was significantly higher than that of AD patients (Fig. 6a, left, black box, and Fig. 6b, *p* = 0.034). In contrast, Aβ_42_Os were observed in AD patients, but not in HC (Fig. 6a, right, red box, and Fig. 6b, *p* = 0.039). In addition, a sandwich ELISA test was performed using the enriched blood samples to evaluate the levels of Aβ_42_Ms and Aβ_42_Os. As shown in Fig. 6c, compared with blood samples from HC, samples from AD patients showed a significant decrease in Aβ_42_Ms (*p* = 0.0265) and an increase in Aβ_42_Os (*p* = 0.086). This phenomenon is consistent with the clinical symptoms reported by multiple studies [28, 66, 68, 69].

To illustrate the ability of the dLFI in Aβ detection, a horizontal comparison of various available technologies in Table 1, such as ELISA [36, 70], nanoparticles-based immunoassays [71], surface-enhanced Raman spectroscopy [39], fluorescence [41], electrochemical biosensors [37], etc. ELISA is mainly used as a stopgap measure with the highest sensitivity in Aβ detection, but it cannot effectively distinguish monomers and oligomers. As shown in Fig. 6d, total Aβ_42_ levels detected by sandwich ELISA using a pair of antibodies recognizing different epitopes of Aβ_42_ showed no significant difference between AD and HC groups (*p* = 0.4837). Considering that the level of soluble Aβ_42_Os in AD patients is elevated, it is easy to be mis-detected in the measurement of Aβ_42_, resulting in the underestimation of Aβ_42_Ms level and poor performance in assessing the progression of AD [36, 72].

**Table 1:** Performance appraisals among Aβ analytical techniques

Method	LOD	Multi-detection	Time	Expertise or devices required		Refs.
ELISA for Aβ_42_	192 pg/mL	No	3.5 h	Yes	[70]	
ELISA for Aβ_42_Os	197 pg/mL	No	3.5 h	Yes	[36]	
Nanoparticles-based immunoassays	163 pg/mL	No	2 h	Yes	[71]	
Surface-enhanced Raman spectroscopy	181 ng/mL	No	NA	Yes	[39]	
Fluorescence	23 ng/mL	No	4 h	Yes	[41]	
Electrochemical biosensors	2.26 ng/mL	No	22 h	Yes	[37]	
dLFI	Aβ_42_Ms: 154 pg/mL Aβ_42_Os: 154 pg/mL	Yes	0.5 h	No	This work	

LOD, limit of detection; ELISA, enzyme-linked immunosorbent assay; dLFI, dual-target lateral flow immunoassay

For nanoparticle-based immunoassays, including surface-enhanced Raman spectroscopy, fluorescence, and electrochemical biosensors, except for lacking the ability to distinguish monomers and oligomers, some special equipment and professional skills are required. On the contrary, dLFI can effectively detect monomers and oligomers by the naked eye within 30 min, which is not only high sensitivity but also simple, user-friendly, and instant detection without special equipment and professional skills. Altogether, our results indicate that dLFI can simultaneously detect Aβ_42_Ms and Aβ_42_Os in patient blood samples, with high sensitivity and specificity (Table 2).

**Table 2:** Correlation of Aβ_42_Os and Aβ_42_Ms levels in blood samples with dLFI test

Samples	Enriched Aβ_42_Ms (ng/mL)	Enriched Aβ_42_Os (ng/mL)	dLFI test
AD1	0.0302	0.3923	−+
AD2	0.0483	0.4273	−+
AD3	0.632	1.482	++
AD4	0.223	0.2452	−+
AD5	0.9104	1.3293	++
AD6	0.6382	0.4382	++
AD7	0.2711	0.2704	++
AD8	0.187	0.2113	++
HC1	0.8204	0.0081	+−
HC2	2.467	0.0080	+−
HC3	0.6502	0.0028	+−
HC4	0.728	0.0023	+−
HC5	0.8213	0.0041	+−
HC6	0.5781	0.0020	+−
HC7	2.683	0.0051	+−

Sandwich ELISA detects the levels of Aβ_42_Ms and Aβ_42_Os in blood samples

AD, Alzheimer’s disease; HC, healthy controls; Aβ_42_Ms, Aβ_42_ monomers; Aβ_42_Os, Aβ_42_ oligomers; dLFI, dual-target lateral-flow immunoassay strip; −+, Aβ_42_Ms negative and Aβ_42_Os positive; ++, Aβ_42_Ms positive and Aβ_42_Os positive; +−, Aβ_42_Ms positive and Aβ_42_Os negative

## Conclusion

This study provided a new method for the simultaneous detection of Aβ_42_Ms and Aβ_42_Os in AD blood using effective and rapid multiple techniques. The dLFI could detect the levels of Aβ_42_Ms and Aβ_42_Os in the blood quickly (within 30 min, including a 25-min enrichment step and a 5-min dLFI test step) and semi-quantitatively by naked eyes. In this system, the pre-incubation step can ensure high sensitivity and stability of the strip sensor. In conclusion, the dLFI allows high-throughput testing of small samples and has the potential to become a powerful tool for the rapid and accurate diagnosis of AD.

## Supplementary Information

**Additional file 1: Table S1.** Information of the participants in this manuscript. **Figure S1**. The HPLC and mass spectrometry results of synthesized Aβ_42_ (**a**, **b**) and Aβ_40_ (**c**, **d**) peptides. **Figure S2**. The HPLC and mass spectrometry results of synthesized p-Tau^396,404^ (**a**, **b**) and p-Tau^231^ (**c**, **d**) peptides. **Figure S3**. The HPLC (**a**) and mass spectrometry (**b**) results of synthesized Cis-Tau peptides. **Figure S4**. Characterization of AuNP–1F12 conjugates. (**a)** The color and UV − vis absorption spectra of synthetic AuNP. (**b)** The Zeta-potentials of AuNP before and after mAb 1F12 modification. (**c)** UV − vis absorption spectra of mAb 1F12 before and after conjugation. The absorption spectrum of different volumes of K_2_CO_3_ (**d**) and concentrations of 1F12 (**e**) for the conjugation of 1F12 with AuNP. Data are presented as means ± SD. **Figure S5**. Characterization of 1F12-modified MNPs. (**a)** The representative SEM image of bare magnetic nanoparticles (MNPs). (Scale bar: 200 nm). (**b)** The principle of synthetic antibody-modified MNPs. The sizes (**c**) and Zeta-potentials (**d**) of MNPs before and after antibody modification. Data are presented as means ± SD.
