# Supplementary material for: Quantitative assessment of AD markers using naked eyes: point-of-care testing with paper-based lateral flow immunoassay
Source: J Nanobiotechnology. 2021 Nov 17;19:366. doi: 10.1186/s12951-021-01111-z (PMC8597216; doi:10.1186/s12951-021-01111-z)
Supplement: Supplementary file 1 — Additional file 1: Table S1. Information of the participants in this manuscript. Figure S1. The HPLC and mass spectrometry results of synthesized Aβ42 (a, b) and Aβ40 (c, d) peptides. Figure S2. The HPLC and mass spectrometry results of synthesized p-Tau396,404 (a, b) and p-Tau231 (c, d) peptides. Figure S3. The HPLC (a) and mass spectrometry (b) results of synthesized Cis-Tau peptides. Figure S4. Characterization of AuNP–1F12 conjugates. (a) The color and UV − vis absorption spectra of synthetic AuNP. (b) The Zeta-potentials of AuNP before and after mAb 1F12 modification. (c) UV − vis absorption spectra of mAb 1F12 before and after conjugation. The absorption spectrum of different volumes of K2CO3 (d) and concentrations of 1F12 (e) for the conjugation of 1F12 with AuNP. Data are presented as means ± SD. Figure S5. Characterization of 1F12-modified MNPs. (a) The representative SEM image of bare magnetic nanoparticles (MNPs). (Scale bar: 200 nm). (b) The principle of synthetic antibody-modified MNPs. The sizes (c) and Zeta-potentials (d) of MNPs before and after antibody modification. Data are presented as means ± SD. [file 12951_2021_1111_MOESM1_ESM.docx]

**Additional file 1**

Table S1. Information of the participants in this manuscript

| Participant | Sex | Age | MOCA | Stage |
| --- | --- | --- | --- | --- |
| AD1 | female | 70 | 10 | Severe |
| AD2 | female | 79 | 12 | Moderate to severe |
| AD3 | female | 77 | 18 | Mild to moderate |
| AD4 | female | 54 | 5 | Severe |
| AD5 | male | 61 | 15 | Mild to moderate |
| AD6 | male | 74 | 13 | Moderate |
| AD7 | male | 63 | 15 | Mild to moderate |
| AD8 | male | 76 | 13 | Moderate |
| Control 1 | female | 73 | 27 | Health |
| Control 2 | female | 57 | 28 | Health |
| Control 3 | female | 55 | 27 | Health |
| Control 4 | male | 70 | 28 | Health |
| Control 5 | male | 55 | 29 | Health |
| Control 6 | male | 64 | 28 | Health |
| Control 7 | male | 74 | 28 | Health |

Abbreviations: AD Alzheimer’s disease, MOCA Montreal cognitive assessment.

**Figures**


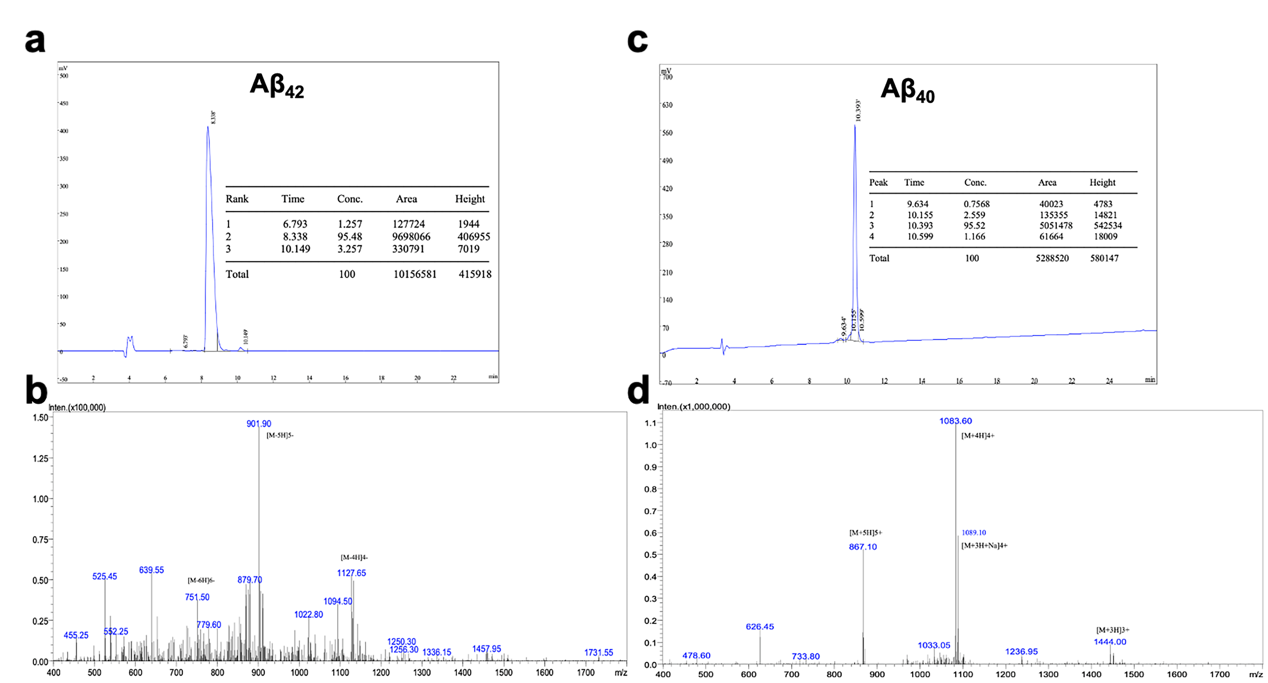


**Figure S1**. The HPLC and mass spectrometry results of synthesized Aβ_42_ (**a**, **b**) and Aβ_40_ (**c**, **d**) peptides.


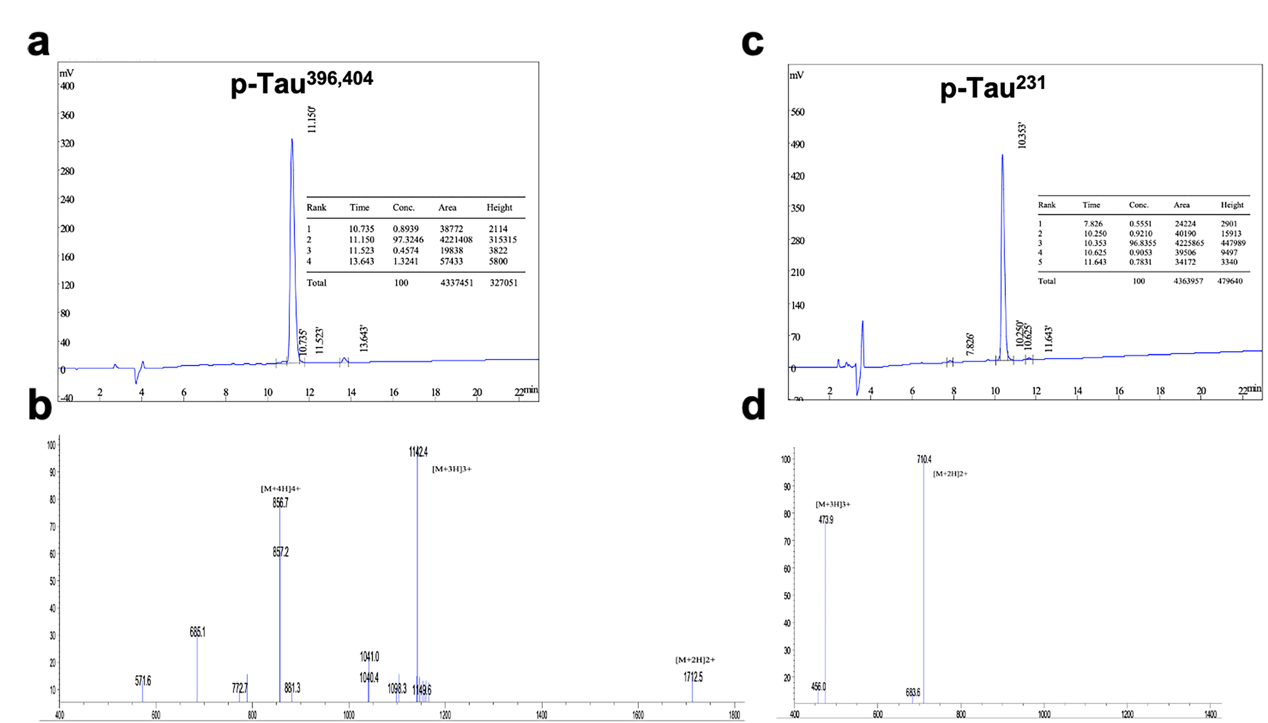


**Figure S2**. The HPLC and mass spectrometry results of synthesized p-Tau^396,404^ (**a**, **b**) and p-Tau^231^ (**c**, **d**) peptides.


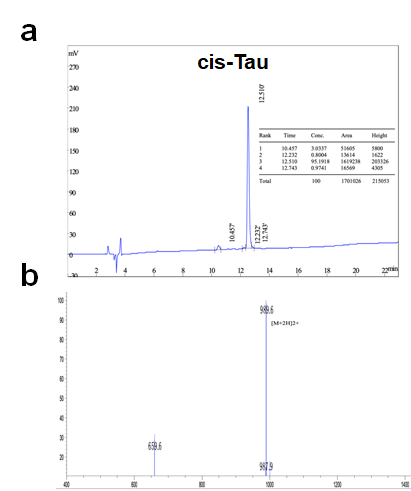


**Figure S3**. The HPLC (**a**) and mass spectrometry (**b**) results of synthesized Cis-Tau peptides.


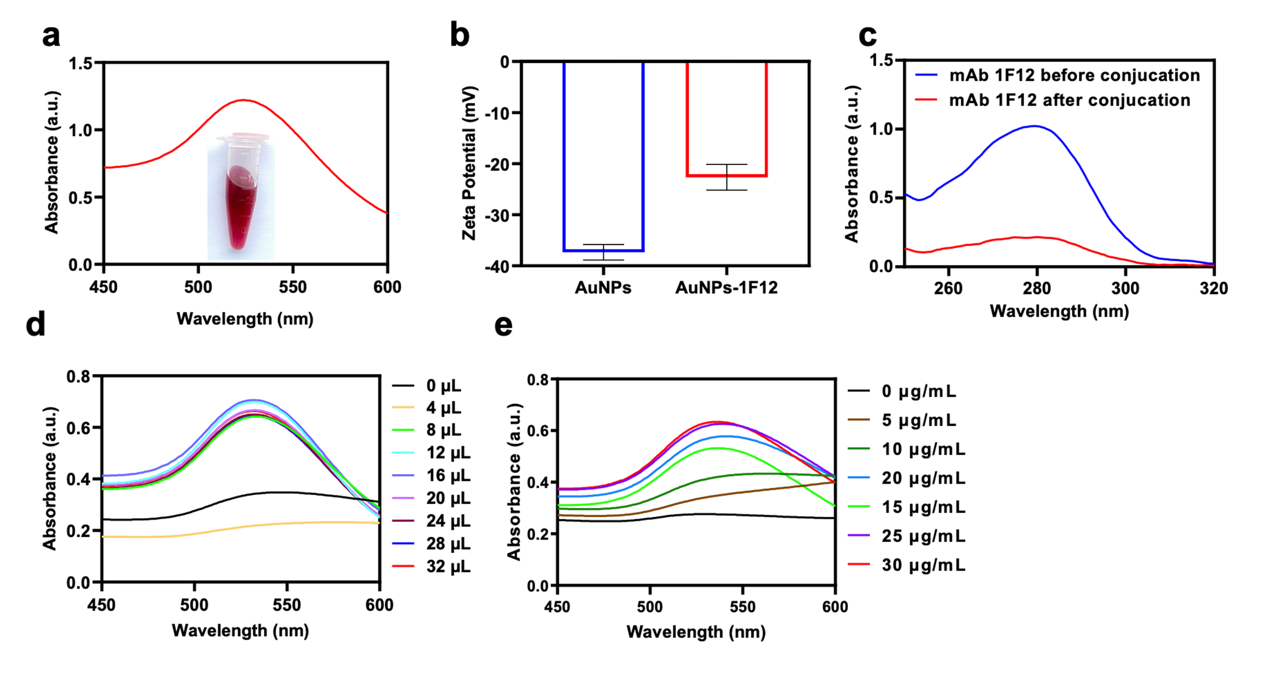


**Figure S4**. Characterization of AuNP–1F12 conjugates. (**a)** The color and UV−vis absorption spectra of synthetic AuNP. (**b)** The Zeta-potentials of AuNP before and after mAb 1F12 modification. (**c)** UV−vis absorption spectra of mAb 1F12 before and after conjugation. The absorption spectrum of different volumes of K_2_CO_3_ (**d**) and concentrations of 1F12 (**e**) for the conjugation of 1F12 with AuNP. Data are presented as means ± SD.

**
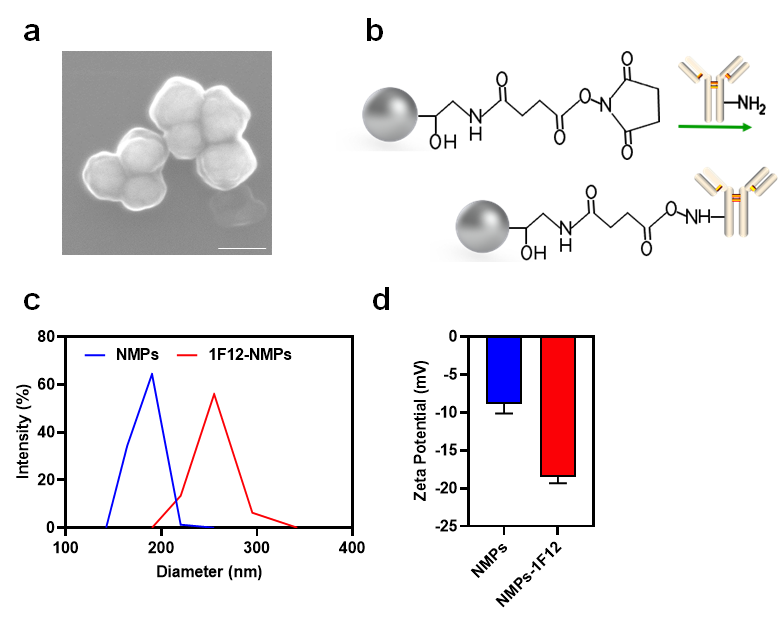
Figure S5**. Characterization of 1F12-modified MNPs. (**a)** The representative SEM image of bare magnetic nanoparticles (MNPs). (Scale bar: 200 nm). (**b)** The principle of synthetic antibody-modified MNPs. The sizes (**c**) and Zeta-potentials (**d**) of MNPs before and after antibody modification. Data are presented as means ± SD.
